# Typification of species names in *Adenocaulon* and *Eriachaenium* (Compositae/Asteraceae, Subfamily Mutisioideae, Tribe Mutisieae, Subtribe Adenocaulinae)

**DOI:** 10.3897/phytokeys.69.9779

**Published:** 2016-09-09

**Authors:** Vicki A. Funk, D. J. Nicholas Hind

**Affiliations:** 1 Department of Botany, NMNH, Smithsonian Institution, P.O. Box 37012, Washington DC, 20013-7012, USA; 2 Herbarium, Royal Botanic Gardens, Kew, Richmond, Surrey TW9 3AE, England, U.K.

**Keywords:** Asteraceae, Composiate, North, Central and South America, Asia, Asia-America disjunct distribution

## Abstract

During the course of a recent research project on *Adenocaulon* and *Eriachaenium* it became apparent that some of the species names had not been typified. In this study we located and designated as much type material as possible for these two genera. We indicate holotypes or lectotypes where appropriate, including one for the type of the genus *Adenocaulon*.

## Introduction

In the process of preparing a manuscript on the phylogenetic placement of *Eriachaenium* Sch. Bip. and *Adenocaulon* Hook. (Funk et al. 2016) it became obvious that many names remained untypified. These two genera have interesting morphologies and distributions but until recently their affinities had never been fully understood (Funk et al. 2016) and the location of the types was not fully resolved. Tracking down the type material required assistance from herbaria in Argentina, Chile, Europe, the UK, and USA (see acknowledgements). We found as much type material as possible and when necessary we provide lectotypifications. No doubt there are isotypes and isolectotypes that we have not located but these should be easier to find with the information we have provided (see Appendix 1).

The species of *Eriachaenium* and *Adenocaulon* are listed below along with their Type citations [! indicates that the specimen was seen by one of the authors and * indicates that it was seen on-line at either JSTOR-GP (continuously updated), the P website (Paris-MNHN, continuously updated), the Virtual Herbaria (continuously updated) or in some cases curators of herbaria sent photos of newly discovered type material. This newly discovered material should be available on JSTOR-GP soon. Dates for collecting trips and potential herbaria that might contain type material were taken from TL-2 (*Taxonomic Literature*, Second edition) and IH II (*Index Herbariorum*, part II) and are cited in the text appropriately. Bittmann (1990a, 1990b) studied the genus *Adenocaulon* and published a detailed study of the morphology and ecology along with a preliminary evaluation of the nomenclature including a description of a new species (Bittmann 1990a).

## Taxonomy

### *Adenocaulon* W.J. Hooker

#### Adenocaulon bicolor

Taxon classificationPlantaeAsteralesAsteraceae

W.J. Hooker

Adenocaulon bicolor W.J. Hooker, Botanical Miscellany 1(1): 19–20. Apr 1829. Plate XV.

##### Syntype material.

[Although the cover page of *Botanical Miscellany* volume 1 lists the publication date as 1830, according to Stafleu and Cowan (1979, page 290) it was published in three parts and part 1 (pages 1–96) was published in 1829]

**Syntype 1.** USA [Washington State], Dense forest of Straits of Juan de Fuca, and near Fort Vancouver and Columbia River, West coast of North America, [1824–1825], *John Scouler s.n.* [1017] [Lectotype: K! here designated; isolectotypes: E00230668*, NY00158065!; OXF* 00005489 (not yet available on JSTOR-GP)]

Scouler’s trip to Northwest USA was in 1824–1825 (Vegter 1986) and the area between the Straits and Fort Vancouver and the Columbia River is in Washington State. The E specimen has “Scouler (1017)” written on it. Vegter (1986) also lists CGE as a possible location for an additional Scouler’s collection but none were found (pers. comm. C. Bartram).

**Syntype 2.** North America, in the Rocky Mountains, [1825–1827], *Thomas Drummond s.n.* [K! (not yet available on JSTOR-GP)]

Lanjouw and Stafleu (1954) reported that Drummond’s collections could also be in A, AWH, B, BM, CGE, DELS, DS, E, FI, G, G-DC, GH, GL, GOET, LD, OXF, P, PH, S, TCD, UC, UMO, UPS, US (not found), W (not found), WU]. They also said that Drummond was in North America 1825–1827 and that he made a second trip 1831–1835 but the latter trip was after the species was described. Stafleu and Cowan (1976) report that the location of Drummond’s original herbarium is unknown but indicate that BM & K have the best sets of his plants.

*Adenocaulon integrifolium* T. Nuttall, Transactions of the American Philosophical Society, New Series 7[1]: 289. 1841. Type material: United States, Oregon, Shady woods of the Wahlamet, near its confluence with the Oregon, [1834], *T. Nuttall, s.n*. [Holotype: GH00000615*; isotypes: K! (not yet available on JSTOR-GP); PH00224269*]

Vegter (1983) listed the date of the expedition as 1834 and said that additional material might have been sent to LIV (not found) and NY (not found). Bittmann (1990a) listed PHIL as the location of an isotype but it appears that this refers to the isotype at PH which was annotated by Bittmann (E. Benamy, pers. com.). According to Stafleu and Cowan (1981, page 786) the prologue was contained in part 1 of volume 7 and published on 12 May 1841.

#### Adenocaulon chilense

Taxon classificationPlantaeAsteralesAsteraceae

E.F. Poeppig ex C.F. Lessing

Adenocaulon chilense E.F. Poeppig ex C.F. Lessing, Linnaea 6: 107–108. 1831.

##### Type material.

Chile. Antuco, [Dec 1828], *E.F. Poeppig 225, diar. 755.* [Holotype most likely at B and destroyed; Lectotype here designated as W* (W1889-0050720); isolectotypes: G00495616*, HAL0110867*, M0029893*, P02505554*, W0064359*, & W0064360*]. Vegter (1983) listed the date of the field trip as 1827–1829 and she listed additional possible locations of herbarium material as: B, BR, CGE, F, FI-W, GOET, K, KIEL, L, LE, LZ, MO, NY, OXF, PR, PRC, US, WRSL.

Lessing’s descriptions seem to be based on specimens that he examined at B. They were most likely collected by B staff/associates or sent to B as a gift/exchange. The Compositae herbarium at B was destroyed during WWII (Hiepko 1987) so when duplicates can be found they are designated as lectotypes. Lessing participated in two series of papers about Compositae in the journal *Linnaea*. The title of Lessing’s first series of papers in *Linnaea* was *Synanthereis Herbarii Regii Berolinensis* (Lessing 1929, 1830a, 1830b, 1930c). The second series included two papers by Lessing (1831a, 1831b), the latter of which included the type description of *Adenocaulon chilense*. These two articles were part of a larger series entitled *De plantis in expeditione speculatoria Romanzoffiana observatis disserere pergunt AD. de Chamisso et D. de Schlechtendal*, and although Chamisso’s original collections were no doubt at LE and Schlechtendal’s at HAL they both probably sent duplicates to B and it is likely that Lessing worked from the duplicate material. Interestingly the “Romanzoffiana” treatment is part of a larger series of papers that began in volume 1 of *Linnaea* (Chamisso 1826). In his preface Chamisso (1826) says that the exploratory expeditions were supported by Count de Romanzoff the Imperial Chancellor in 1815, hence the name of the article. The preface mentions other collectors but does not mention Poeppig. Poeppig’s original set is supposed to be at W (Vegter 1983) and it is likely that a duplicate was sent to B. There are two collections at W (E. Vitek, pers. comm.) and one has been selected as the lectotype.

There is some confusion as to the collecting number: on many specimens there are two numbers, the number of the printed labels differing somewhat from the “diario” numbers that are often handwritten. As a result, some records for this particular collection have the number 755 but others list 255 (likewise JSTOR-GP entries list one or the other). Some specimens clearly have both 755 & 225 printed on the labels and others do not. It is possible that the collecting number is 755 as it appears handwritten on the lectotype (and on the printed lables as “diar. 755”) and other type material has 255 printed on the labels and this may be a species number. Vitek (pers. comm.) provided an example of how the collecting number might be cited and we have followed that.

*Adenocaulon lechleri* C.H. Schultz Bipontinus, Flora 38: 113. 1855. Type material. Chile. Ad portum Port Famine, s.d. [1850–1853], *W. Lechler 1245* [Holotype: P02505548* ex Herb. Sch. Bip.; isotypes, BR0000005630134*, G00222157*, G00222158*, G00222159*, GH00000617*, M0029894*, M0029895*, NY00158067*, P02505546*, P02505547*, P03313093*]. On JSTOR-GP there is specimen from K from *Herbarium Hookerianum* collected by Lechler (s.n.) but with a date of 20 January 1854 and a different location. This is not believed to be type material. Chaudhri et al. (1972) gave the dates of this trip as 1850–1853.

*Boerhavia nudicaulis* R.A. Philippi, Linnaea 29: 37. 1857. Type material. Chile. Chillán, 1 Dec 1855, *P. Germain s.n* [Holotype: SGO000001680*; isotypes: G00414903*; K000500419!. K000500419 is not listed as a type in JSTOR-GP and the date printed on the label is ‘1856 et 1857”, but it has the correct location and was originally identified as *Boerhavia nudicaulis* so it is probably an isotype.]

According to Lanjouw and Stafleu (1957), Germain was in Chile 1854–1890 and they indicated that there may be additional specimens at BM, F, FI, P, W and Philippi types may be at BAF (no reply).

#### Adenocaulon himalaicum

Taxon classificationPlantaeAsteralesAsteraceae

M.P. Edgeworth

Adenocaulon himalaicum M.P. Edgeworth, Transactions of the Linnean Society of London 20: 64. 1851.

##### Type material.

Himalaya in sylvis, 7000–9000 ft, between Nagkanda and Kotgurh [India, Himachal Pradesh], 1844, *M.P. Edgeworth 15* [Holotype: K000250901!; Probable isotypes: K! K000250901, OXF* (OXF00005487, not yet available in JSTOR-GP)].

The printed label on the Holotype has a date of 1844, the OXF sheet has the handwritten date of Oct 1840, and the K isotype has no date. TL2 (Stafleu and Cowan 1976 and Lanjouw and Stafleu 1957) indicated that an additional specimen maybe at G-DC (not found, pers. comm. Loze). Only the K specimen has a printed label with a date and a “collecting number” so these may have been added by Kew. Since dates on these older specimens are often mixed up it seems likely that the second K specimen and the OXF specimen are isotypes.

#### Adenocaulon adhaerescens

Taxon classificationPlantaeAsteralesAsteraceae

C.J. Maximowicz

Adenocaulon adhaerescens C.J. Maximowicz, Primitiae florae Amurensis: 152–154. 1859.

##### Type material.

Russia, Amur, [7 July 1855], *C.J. Maximowicz s.n.*

Amur is an area in Siberia (Russia) near the border with China. Vegter (1976) indicated that Maximowicz went to Amur in 1854–1856 and again in 1859–1860 but the latter years are too late for this species description. The lectotype selected below has the date of 7 July 1855 on the label.

According to the protologue there are five syntypes all collected by Maximowicz from Amur, the Khabarovsk Region of Russia. Maximowicz worked at LE and so the material he used to describe this species should be there.

**Syntype 1.** Borbi (an Nadelholzrändern, 27 June fl. pr.) [Borbi, Russia: 51.24, 139.36; 14 Km SSE of Tsimmermanovka and the Amur River (Anonymous 1864)]

**Syntype 2.** Ussuri-Mündung [mouth of the Ussuri River], Wäldern, and especially frequent in Waldstegen, Poddale, 20 May (sterile)

**Syntype 3.** Chungar [Khungari River], 11 July (fl. pr.) Lectotype here designated: LE01013892* (not yet available in JSTOR-GP); Isolectotypes: H1023222*, K000768768!, S0940878*)

**Syntype 4.** Dshare [mouth of “Dondon” (now Anyuy) River], 18 July (flor. et frf.)

**Syntype 5.** Ssargu [now Sharga Lake], 14 July 1855 (fl. et defl.)

Syntype 3 was selected as the lectotype because the location, day and month are the same as those listed in the protologue. All other specimens either had no information on the actual sheet or conflicting information. Vegter (1976) listed other herbaria that might hold type material: L, NY, PU, W.

#### Adenocaulon lyratum

Taxon classificationPlantaeAsteralesAsteraceae

S.F. Blake

Adenocaulon lyratum S.F. Blake, Journal of the Washington Academy of Sciences 24: 435–436. 1934.

##### Type material.

Guatemala. In open woods, Chichavac, Dept. Chimaltenango, alt. 2530 m, 20 Sept 1933, *A.F. Skutch 622*, [Holotype: US00128315!; Isotypes: CAS0000062!; GH00000616*; K000500423*; LL00374377*; MICH1108850*]

#### Adenocaulon nepalense

Taxon classificationPlantaeAsteralesAsteraceae

M. Bittmann

Adenocaulon nepalense M. Bittmann, Candollea 45: 403–405. 1990.

##### Type material.

Nepal, Jaljale, 3400 m, 22 Aug 1984, *M. Farille & G. Lachard 847509* [Holotype: GOET000995*; isotype: G (as cited by Bittman 1990a; not found in the G herbarium, pers.comm. L. Gautier)]

### *Eriachaenium* C.H. Schultz Bipontinus

#### Eriachaenium magellanicum

Taxon classificationPlantaeAsteralesAsteraceae

C.H. Schultz Bipontinus

Eriachaenium magellanicum C.H. Schultz Bipontinus, Flora 38: 120–121. 1855.

##### Type material.

Chile. Prov. Magallanes: Oazy Harbour, s.d. [1850–1853], *W. Lechler 1256* [Holotype: P04388712* ex Herb. Sch. Bip.; isotypes: K000500417!, K000500418!, P04388711*, S10-34491*]

Chaudhri et al. (1972) list the dates of this trip as 1850–1853.

## Supplementary Material

XML Treatment for Adenocaulon bicolor

XML Treatment for Adenocaulon chilense

XML Treatment for Adenocaulon himalaicum

XML Treatment for Adenocaulon adhaerescens

XML Treatment for Adenocaulon lyratum

XML Treatment for Adenocaulon nepalense

XML Treatment for Eriachaenium magellanicum

## Appendix 1

TYPE collections [collector(s) and number, date collected, basionym, current species name]

*Drummond T., s.n.*, [1925–1927], ***Adenocaulon bicolor*** WJ Hooker

*Edgeworth M.P., 15*, 1844, ***Adenocaulon himalaicum*** Edgeworth

*Farille M. & Lachard G. 847509*, 22 Aug 1984, ***Adenocaulon nepalense*** M Bittmann

*Germain P., s.n.*, 1 Dec 1855, *Boerhavia nudicaulis* Phil. = ***Adenocaulon chilense*** Lessing

*Lechler W., 1245*, [1850–1853], *Adenocaulon lechleri* Sch. Bip. = ***Adenocaulon chilense*** Lessing

*Lechler W., 1256*, [1850–1853], ***Eriachaenium magellanicum*** Sch. Bip.

*Maximowicz C.J., s.n.*, s.d. [1854–1856], *Adenocaulon adhaerescens* Maximowicz = ***Adenocaulon himalaicum*** Edgeworth

*Nuttall T., s.n.*, s.d. [1834], *Adenocaulon integrifolium* T Nuttall = ***Adenocaulon bicolor*** WJ Hooker

*Poeppig E.F., 225 and/or 755*, s.d. [1827–1829], ***Adenocaulon chilense*** Lessing

*Scouler J., s.n*, s.d. [1824–1825], ***Adenocaulon bicolor*** JD Hooker

*Skutch A.F., 622*, 20 Sep 1933, ***Adenocaulon lyratum*** SF Blake
